# Vitamin D_3_ alters macrophage phenotype and endosomal trafficking markers in dairy cattle naturally infected with *Mycobacterium avium* subsp. *paratuberculosis*

**DOI:** 10.3389/fcimb.2022.1021657

**Published:** 2022-10-05

**Authors:** Taylor L. T. Wherry, Rohana P. Dassanayake, John P. Bannantine, Shankumar Mooyottu, Judith R. Stabel

**Affiliations:** ^1^ Infectious Bacterial Diseases, National Animal Disease Center, United States Department of Agriculture - Agricultural Research Service (USDA-ARS), Ames, IA, United States; ^2^ Department of Veterinary Pathology, College of Veterinary Medicine, Iowa State University, Ames, IA, United States; ^3^ Ruminant Diseases and Immunology, National Animal Disease Center, United States Department of Agriculture - Agricultural Research Service (USDA-ARS), Ames, IA, United States

**Keywords:** *Mycobacterium avium* subsp. *paratuberculosis*, cattle, vitamin D, macrophage, Rab5, Rab7, CD80, CD163

## Abstract

Macrophages are important host defense cells in ruminant paratuberculosis (Johne’s Disease; JD), a chronic enteritis caused by *Mycobacterium avium* subsp. *paratuberculosis* (MAP). Classical macrophage functions of pathogen trafficking, degradation, and antigen presentation are interrupted in mycobacterial infection. Immunologic stimulation by 25-hydroxyvitamin D_3_ (25(OH)D_3_) and 1,25-dihydroxyvitamin D_3_ (1,25(OH)_2_D_3_) enhances bovine macrophage function. The present study aimed to investigate the role of vitamin D_3_ on macrophage phenotype and endosomal trafficking of MAP in monocyte-derived macrophages (MDMs) cultured from JD-, JD+ subclinical, and JD+ clinically infected cattle. MDMs were pre-treated 100 ng/ml 25(OH)D_3_ or 4 ng/ml 1,25(OH)_2_D_3_ and incubated 24 hrs with MAP at 10:1 multiplicity of infection (MOI). *In vitro* MAP infection upregulated pro-inflammatory (M1) CD80 and downregulated resolution/repair (M2) CD163. Vitamin D_3_ generally decreased CD80 and increased CD163 expression. Furthermore, early endosomal marker Rab5 was upregulated 140× across all stages of paratuberculosis infection following *in vitro* MAP infection; however, Rab5 was reduced in MAP-activated MDMs from JD+ subclinical and JD+ clinical cows compared to healthy controls. Rab7 expression decreased in control and clinical cows following MDM infection with MAP. Both forms of vitamin D_3_ reduced Rab5 expression in infected MDMs from JD- control cows, while 1,25(OH)_2_D_3_ decreased Rab7 expression in JD- and JD+ subclinical animals regardless of MAP infection *in vitro*. Vitamin D_3_ promoted phagocytosis in MDMs from JD- and JD+ clinical cows treated with either vitamin D_3_ analog. Results from this study show exogenous vitamin D_3_ influences macrophage M1/M2 polarization and Rab GTPase expression within MDM culture.

## 1 Introduction

Paratuberculosis, commonly known as Johne’s Disease (JD), is a chronic enteritis of ruminants caused by the intracellular pathogen, *Mycobacterium avium* subsp. *paratuberculosis* (MAP). Initially, MAP is transmitted through the fecal-oral route in young calves, with infection and disease susceptibility typically decreasing with age (Windsor and Whittington, 2010). Other significant contributing factors to MAP susceptibility include cattle breed and genetic polymorphisms related to immune function (Bhide et al., 2009; Mucha et al., 2009; Pinedo et al., 2009; Sorge et al., 2011). Propagation of disease manifests through a chronic, asymptomatic subclinical phase, characterized by cell mediated pro-inflammatory Th1 responses, namely IFN-γ, that contribute to mechanisms of bacterial control and prevent progression to clinical disease (Stabel, 1996; Stabel, 2000). Transition to the clinical stage involves impairment and loss of protective pro-inflammatory responses, resulting in a humoral anti-inflammatory Th2 cytokine profile represented by increased IL-10 secretion (Hussain et al., 2016). This stage is also often accompanied by the production of MAP-specific antibodies that do not significantly contribute to disease resolution but are a useful diagnostic tool (Whitlock et al., 2000). Clinical disease results in a malnourished animal with decreased economic contribution in terms of milk production and trade value. Herd intervention methods largely rely on good animal husbandry practices, as MAP can persist for lengthy periods of time in the environment and there are currently no commercially available options for treatment (Whittington et al., 2004; Whittington et al., 2005; Eppleston et al., 2014). A major factor in the lack of effective vaccines and therapeutics against MAP infection is the stealthy nature of this pathogen and its mastery in evading the immune system.

Upon traversing the intestinal epithelium, MAP targets its preferential host reservoir, the resident tissue macrophage (Bannantine and Bermudez, 2013). Efficiency of host macrophage signaling and its interactions with key subpopulations of T lymphocytes is essential in determining the fate of intracellular MAP (Chiodini and Davis, 1993; Koets et al., 2002). As an initial defense mechanism, the innate immune response involves processing pathogens through the endocytic pathway within phagocytic cells. Following uptake of the pathogen into a phagosome, this compartment undergoes a transitional process of maturation through fusion with early and late endosomes under normal homeostatic conditions. Phagosomal membrane acquisition of Rab5 is an early endosome identifier. Recruitment of Rab7, a defining marker between early and late endosome phases, leads to the gradual replacement of Rab5. Downstream consequences of this marker transition leads to endosome/phagosome and lysosome fusion through GTP bound and activated Rab7 binding Rab7-interacting lysosomal protein (RILP) and the resulting interactions with lysosome associated membrane protein 1 and 2 (LAMP-1 and LAMP-2) (Clemens and Horwitz, 1995; Cantalupo et al., 2001; Harrison et al., 2003). These events lead to acidification of the compartment, with the ensuing killing and degradation of the pathogen.

MAP is able to survive and replicate within the macrophage by arresting phagosome maturation, demonstrating persistence for up to 15 days (Kuehnel et al., 2001). Other intracellular pathogens possess similar survival strategies that involve suspending phagosome maturation and its resulting acidification. *Legionella pneumophila*, for example, survives by preventing initial interaction with endosomes and instead hijacks exit trafficking vesicles from the endosomal reticulum (Swanson and Isberg, 1995; Kagan and Roy, 2002). Mouse macrophage RAW 264.7 cells infected with *Mycobacterium bovis* (*M. bovis*) BCG have been shown to maintain Rab7 in a GDP-bound inactive form (Sun et al., 2007). Furthermore, *Mycobacterium tuberculosis* (*M. tb*) ultimately subverts host defenses by inhibiting the function of late endosome markers through blocking fusion events between Rab5 and Rab7 signaling (Clemens et al., 2000a; Rankine-Wilson et al., 2021). *M. tb* containing phagosomes merge to an extent with early endosomes and gain Rab5 (Clemens et al., 2000a), which resides transiently on the endosomal membrane (Vieira et al., 2003). However, cell wall mannosylated lipoarabinomannan (ManLAM) in *M. tb* interrupts this step in the pathway by inhibiting phosphatidylinositol 3-phosphate (PI3P) signaling (Fratti et al., 2001; Fratti et al., 2003; Purdy et al., 2005). PI3P functions to bind early endosome autoantigen (EEA1), which then facilitates early endosome tethering to the phagosome and their subsequent fusion (Christoforidis et al., 1999). As a result, *M. tb* can negatively regulate Rab5 acquisition and downstream effectors. Additionally, *M. tb* can interrupt signaling events for late endosomes and phagosomes by blocking Rab7-RILP binding, resulting in reduced acquisition of LAMP-1 (Clemens et al., 2000b).

There is much interest in the role that vitamin D_3_ plays in modulating immune system responses to pathogen infection, with extensive work having been done in human tuberculosis (Crowle et al., 1987; Liu et al., 2006; Liu et al., 2007). These non-classical effects of vitamin D_3_ are achieved through conversion of circulating 25-dihydroxyvitamin D_3_ (25(OH)D_3_) to its bioactive form, 1α-dihydroxyvitamin D_3_ (1,25(OH)_2_D_3_) locally in immune cells (Adams et al., 1983; Nelson et al., 2010b). Recent efforts have been made to further characterize the dynamics between vitamin D_3_ immunomodulation and mycobacterial infection in cattle. It is important to note that JD+ clinical cows have significantly reduced circulating 25(OH)D_3_ compared to healthy cows (Sorge et al., 2013; Stabel et al., 2019; Wherry et al., 2022a). Recent reports have also associated diffuse paratuberculosis lesions, often accompanied with clinical disease, with significantly reduced serum cholesterol levels (González et al., 2005; Canive et al., 2021). This may highlight a need to evaluate vitamin D_3_ supplementation practices in the industry. Additionally, serum levels of 25(OH)D_3_ >80 ng/ml have shown to reduce cattle susceptibility to *M. bovis* infection, whereas this benefit is lost if levels reach <45 ng/ml (López-Constantino et al., 2022). Our lab has reported 1,25(OH)_2_D_3_ treatment of MAP infected monocyte-derived macrophages (MDMs) cultured with PBMCs results in increased secretion of pro-inflammatory cytokines IL-1β and IL-12A, with a concomitant decrease of anti-inflammatory IL-10 secretion (Wherry et al., 2022a). That study also observed upregulated phagocytosis of MAP in MDMs from clinically infected cows. *M. bovis* infected MDMs treated with 1,25(OH)_2_D_3_ show robust nitric oxide production and upregulated inducible nitric oxide synthase (*NOS2*) gene expression (García-Barragán et al., 2018). Additionally, PBMCs from *M. bovis* BCG vaccinated cattle treated with 1,25(OH)_2_D_3_ or 25(OH)D_3_ observe antigen-specific increases of nitric oxide along with a reduction in IFN-γ production (Nelson et al., 2011).

Mechanisms of immune evasion by MAP in bovine paratuberculosis remain to be fully elucidated. Consequences of vitamin D_3_ regulation on immune system signaling add further complexity to the dynamic interplay of the pathogen and host. The primary objective in this study was to characterize the effects of vitamin D_3_ on phenotypic changes and endosomal maturation within MDMs cultured from cattle at different stages of paratuberculosis. We hypothesized that vitamin D_3_ treatment would elicit changes in macrophage phenotype receptor expression that would be functionally represented by changes in endosomal maturation marker expression and intracellular MAP viability. To the best of our knowledge, this is the first study to investigate modulation of endosomal markers by vitamin D_3_ in cattle infected with MAP.

## 2 Materials and methods

### 2.1 Animals

Holstein dairy cows aged 3 – 14 years were used in this study. To prevent cross-contamination, animals were housed separately on-site according to positive or negative infection status with MAP. Cattle in the JD+ groups were all naturally infected with MAP. All experimental procedures were approved by the IACUC (National Animal Disease Center, Ames, IA). Housing facilities are accredited by the American Association for Accreditation of Laboratory Animal Care.

Diagnostic tests measuring serum MAP-specific antibody levels (Herdchek; IDEXX, Westbrook, ME), bovine IFN-γ plasma levels (Bovigam; Prionics, La Vista, NE), and fecal shedding detected by culture on Herrold’s egg yolk medium (Becton Dickinson, Sparks, MD) were used to categorize cows by stage of MAP infection, as previously described (Stabel and Bannantine, 2019). JD+ clinical cows (n=7) were ELISA positive for MAP serum antibody, with an average S/P ratio of 1.27. The average MAP-specific IFN-γ recall response for this group was OD_450_ 0.39 ± 0.17 (Abs_450nm_MPS-Abs_450nm_NS). These animals were also culture positive for MAP, having an average of 189 CFU/g fecal matter. JD+ subclinical cows (n=7) were defined as ELISA negative for MAP serum antibodies and had an average IFN-γ OD_450_ of 0.26 ± 0.11. Three cows from this group were fecal culture positive for MAP and had an average shedding value of 12 CFU/g fecal matter. JD- control cows (n=9) were negative for all MAP diagnostic tests.

### 2.2 Bacterial culture

This study utilized a low-passage virulent strain of MAP (cow 167; NADC, Ames, IA) isolated from a dairy cow in the clinical stage of Johne’s disease. MAP 167 cultures were prepared by inoculating a previously frozen aliquot into 450 ml of Middlebrook 7H9 broth (Becton Dickinson, Franklin Lakes, NJ) at pH 5.9, supplemented with 1 mg mycobactin J at 2 mg/ml (Allied Monitor Inc., Fayette, MO), 50 ml oleic acid-albumin-dextrose complex (OADC; Becton Dickinson), and 0.05% Tween 80 (Sigma-Aldrich, St. Louis, MO). Bacterial cultures were incubated at 39°C until they reached the logarithmic growth phase at a 540 nm optical density (OD_540_) of 0.2 to 0.4. Aliquots were prepared and stored in Dulbecco’s Phosphate Buffered Saline (D-PBS) (Sigma-Aldrich) as described previously (Paustian et al., 2008; Stabel et al., 2012; Bradner et al., 2013).

Control cultures included a laboratory MAP K10-green fluorescent protein (GFP) strain and non-pathogenic *M. smegmati*s. MAP K10-GFP was grown in the same conditions and the same media as MAP 167 with the addition of 50 μg/ml kanamycin. *M. smegmatis* was cultured in tryptone, glucose, and yeast extract (TGY) media in Erlenmeyer shaker flasks incubated at 39°C with agitation for 12 hrs to log phase. All cultures were washed and resuspended in D-PBS.

### 2.3 Vitamin D_3_ stock preparation

25(OH)D_3_ and 1,25(OH)_2_D_3_ stocks were prepared in pure ethanol and supplied by Dr. T. A. Reinhardt (NADC). Working stocks of 25(OH)D_3_ and 1,25(OH)_2_D_3_ were made by diluting in 100% fetal bovine serum to a concentration of 1,000 ng/ml and 40 ng/ml, respectively. Following a final 1:10 dilution, cell culture treatment wells had a final concentration of 10% FBS with either 100 ng/ml 25(OH)D_3_ or 4 ng/ml 1,25(OH)_2_D_3_. Vitamin D_3_ treatment concentrations used in these experiments were selected based on previous work in cattle (Nelson et al., 2010b; Nelson et al., 2011). Final ethanol concentrations for 25(OH)D_3_ and 1,25(OH)_2_D_3_ treatments did not exceed 0.004% or 0.031%, respectively. All stocks were stored in airtight glass vials at -20°C and kept protected from light at all times.

### 2.4 PBMC isolation and MDM culture

PBMCs were isolated from whole blood drawn from jugular venipuncture into 2× acid-citrate-dextrose (in-house, 1:10). Whole blood diluted 1:2 in D-PBS was centrifuged 800 × g for 30 min and the resulting buffy coat fraction was laid over Histopaque-1077 (Sigma-Aldrich) for density centrifugation. PBMCs underwent lysing-restoring steps to remove red blood cell contamination, washed in D-PBS, and resuspended in complete growth medium [cRPMI; RPMI-1640 with L-glutamine and HEPES (Gibco, Grand Island, NY), 1% antibiotic-antimycotic (100 U/ml penicillin, 100 μg/ml streptomycin, 250 ng/ml Amphotericin B, Gibco), 1% MEM non-essential amino acids solution (100×, Gibco), 2% MEM essential amino acids solution (50×, Gibco), 2 mM L-glutamine [200 mM, Gibco]; 1% sodium pyruvate (100mM, Gibco); and 50 μM 2-mercaptoethanol (50 mM, Gibco)] supplemented with 10% (v/v) heat inactivated fetal bovine serum (FBS Defined; HyClone Cytiva, Marlborough, MA). Live cells were counted using trypan blue dye exclusion on a TC20 automated cell counter (Bio-Rad, Hercules, CA). Densities of the unfractionated PBMCs were adjusted to 4.0 × 10^6^ cells per ml in cRPMI with 10% FBS and seeded at 1 ml onto 24 well ibi-treat μ-plates (Ibidi, Fitchburg, WI) for fluorescently labeling cellular targets or 24 well flat-bottom plates (Becton Dickinson, Franklin Lakes, NJ) for cell counting purposes. The unfractionated PBMCs were incubated 5-6 days in a 39°C humidified incubator to generate MDMs. FBS used in all experiments was heat inactivated and filtered through a 0.2 μm filter. Similar long-term culture of unfractionated PBMCs, which contain lymphocytes to promote survival in long-term culture, have been described by our lab previously (Khalifeh and Stabel, 2004; Dassanayake et al., 2021; Wherry et al., 2022a).

### 2.5 Vitamin D_3_ treatment and MAP inoculation

Cell culture media was replaced on day 5 to pre-treat with 1 ml cRPMI containing 10% FBS and 100 ng/ml 25(OH)D_3_ for 24 hrs. Untreated control replicates also had cell culture medium replaced on this day with 1 ml cRPMI containing 10% FBS only. On day 6, another set of replicate wells were pre-treated with 4 ng/ml 1,25(OH)_2_D_3_ for 6 hrs. During this incubation, separate culture plates designated for counting MDMs were gently washed twice with 1 ml room temperature D-PBS to remove nonadherent cells, then incubated on ice for 15 min after addition of 1 ml cold D-PBS to promote non-mechanical detachment of adherent cells. Detached MDMs were then removed from culture plates and counted using trypan blue dye exclusion on a TC20 automated cell counter. Completion of vitamin D_3_ pre-treatments were timed to intersect on day 6. At this time, all wells had fresh media replaced with their respective treatments for the remaining adherent cells, and those assigned for MAP infection were inoculated at a 10:1 MOI +/- 25(OH)D_3_ or 1,25(OH)_2_D_3_ in 1 ml cRPMI with 10% FBS. MAP K10-GFP and *M. smegmatis* treatments were also inoculated on day 6 of culture at a 10:1 MOI. All plates were then incubated 24 hrs at 39°C.

Cell morphology of adherent cells was evaluated following washing and was observed to be consistently elongated/stellate in nature across all cultures, consistent with characteristics of macrophages. This step was necessary to evaluate the possibility of eosinophil contamination. Furthermore, >80% of cells in culture expressed CD163, which is an exclusive marker to monocytes/macrophages. An absence of CD163 expression does not rule out the cell being a macrophage, and thus the authors believe there is good evidence the methods described herein yielded a high purity of macrophages in culture and eosinophil contamination was negligible.

### 2.6 Immunocytochemistry

#### 2.6.1 Macrophage phenotype and vitamin D receptor expression

M1/CD80 (BOV2107; Washington State University, Pullman, WA) and M2/CD163 (BOV2141; Washington State University, Pullman, WA) antibodies were purified using a mouse specific antibody purification column system (ab128749; Abcam, Waltham, MA) and directly conjugated to AF488 and AF594, respectively, using Lightning-Link Alexa Fluor labeling kits (Abcam). MDMs cultured in coverslip-bottom 24 well plates (Ibidi) were first washed twice with 0.02M MOPS/150mM NaCl/1 mM MgCl_2_ (wash buffer) and nonspecific antibody labeling was blocked using serum-free blocking buffer (X0909, Agilent Technologies, Santa Clara, CA) for 30 min at room temperature. Extracellular targets CD80 and CD163 were labeled with directly conjugated antibodies diluted in wash buffer and incubated in the dark at room temperature for 1 hr. Cells were washed then fixed with paraformaldehyde diluted to 1% (157-4; Electron Microscopy Sciences, Hatfield, PA) for 15 min, washed, and permeabilized with 0.02M MOPS/150mM NaCl/1 mM MgCl_2_ containing 0.1% saponin (perm buffer) three times for 5 min each in preparation for labeling intracellular targets. Cells were blocked again for 30 min and then incubated with primary antibody to vitamin D receptor (VDR) (LS-C407668; LSBio, Seattle, WA) followed by AF647 goat anti-rabbit IgG (A21244; Invitrogen, Carlsbad, CA), both diluted in perm buffer and incubated for 1 hr each. Cells were counter-stained with 1 μg/ml DAPI for 5 min, washed, and approximately 250 μl of non-hardening mounting medium (50001; Ibidi) was added to each well for imaging. All targets were measured within MDMs, which were identified through creating MDM boundaries (regions of interest; ROI) within the Nikon NIS-Elements software by superimposing and combining binary layers for M1 and M2 macrophage markers to capture the entire population.

#### 2.6.2 Endosomal markers

Rab5 (ab13253; Abcam) and Rab7 (PA5-52369; Invitrogen) intracellular targets were labeled in separate sets of cell cultures. MDMs were washed, fixed, blocked, and permeabilized as detailed above. Rab5 and Rab7 primary antibodies were each diluted with CD68 primary antibody (M0718; Agilent Technologies, Santa Clara, CA) in perm buffer and incubated with MDMs in the dark at room temperature for 1 hr. Following washing with perm buffer, secondary antibodies targeted to Rab5 (AF594, A11072; Invitrogen) or Rab7 (AF647, A21244; Invitrogen) along with CD68 (AF488, A11029; Invitrogen) were diluted in perm buffer and added to MDM cultures to incubate 1 hr in the dark at room temperature. MDMs were counter stained and prepared for imaging as described above.

MAP K10-GFP and *M. smegmatis* infected MDM cultures followed the above Rab5 and Rab7 labeling protocols with one alteration each. In the Rab5 protocol, CD68 primary antibody was coupled with an AF647 secondary antibody (115-605-205; Jackson Labs, West Grove, PA) and in the Rab7 protocol, CD68 primary antibody was coupled with an AF594 secondary antibody (115-585-205; Jackson Labs). MAP K10-GFP was detected on the 488 nm laser while *M. smegmatis* was also detected on the 488 nm laser after labeling with SYTO 9 diluted in perm buffer and incubated for 15 minutes in the dark. All targets were measured within CD68+ MDMs.

#### 2.6.3 LysoTracker assay

Lysosomal acidification was detected using LysoTracker Red DND-99 (L7528; Molecular Probes, Eugene, OR). MDMs were incubated with 200 μl LysoTracker diluted to 75 nM in cRPMI with 10% FBS for 1 hr at 39°C. Steps for cell fixation, blocking, and permeabilization were performed as detailed above. CD68 primary antibody was then added, followed by an AF647 secondary antibody (115-605-205; Jackson Labs) and cells were counter stained with 1 μg/ml DAPI for 5 min. Approximately 250 μl of non-hardening mounting medium (Ibidi) was added to each well in preparation for imaging. As previously described for the Rab5 and Rab7 protocols, GFP expression or SYTO 9 staining of mycobacteria were detected on the 488 nm laser channel through GFP or SYTO 9 fluorescence. All primary and secondary antibodies were incubated with MDM culture for 1 hr at room temperature in the dark. LysoTracker was measured within MDMs, which were identified through CD68 expression.

#### 2.6.4 Intracellular MAP viability assay

The viability of intracellular MAP taken up by MDMs was assessed using the *Bac*Light viability assay. MDMs were washed with wash buffer and an anti-MAP rabbit polyclonal (#272, in-house) antibody was added to identify and exclude any extracellular MAP. This primary antibody was labeled with an Alexa Fluor 405 (AF405) goat-anti rabbit secondary antibody (A31556; Invitrogen). SYTO 9 (488 nm excitation) and P.I. (561 nm excitation) are fluorescent nucleic acid dyes supplied in the *Bac*Light viability kit (L7012; Life Technologies, Carlsbad, CA). Each dye was diluted according to the manufacturer’s recommendations in perm buffer. Saponin detergent in this buffer permeabilized cells and facilitated intracellular staining of MAP through entry of P.I. into the live bovine MDMs. MDMs were incubated in 0.5 ml of *Bac*Light dye solution for 15 min at room temperature in the dark. Finally, prior to live cell imaging on the confocal microscope the viability dye was removed, and 1 ml of wash buffer was added.

P.I. is membrane impermeant and exhibits a stronger binding affinity for nucleic acids than STYTO 9 (Stocks, 2004); therefore, permeabilization of the live bovine MDMs resulted in fluorescent labeling of the nuclei with P.I. Size limits were applied in the 488 nm and 561 nm channel to treat equally the channels measuring intracellular MAP. Setting size parameters allowed for exclusion of large mammalian nuclei labeled with P.I. and quantification of MAP only. Additionally, MAP co-labeling with P.I. or SYTO 9 and AF405 secondary antibody signal were identified as extracellular and excluded from the intracellular MAP viability calculation (area μm^2^). Average live and dead MAP area were calculated per image by the software measuring binary area pixels expressing fluorescence in the 488 nm and 561 nm channels, respectively.

### 2.7 Confocal microscopy

Images were acquired with a 40× Nikon Plan Fluor N.A. 1.3 objective using oil immersion with 6.2 second pixel dwell time on a Nikon A1 Resonance Plus confocal microscope using NIS-Elements Advanced Research software v5.11 (Nikon, Melville, NY). The instrument contains a 4-laser gallium-arsenide-phosphide/normal photomultiplier tube (GaAsP PMT) fluorescence detector unit (A1-DU4) with two GaAsP PMTs (488 and 561 nm) and two normal PMTs (405 and 640). Fluorescent signal was detected sequentially using the following solid-state diode lasers and bandpass filters: 405 nm (450/50 nm), 488 nm (525/50 nm), 561 nm (600/50 nm), and 640 nm (685/70 nm). A minimum of 10 images were acquired per cow and treatment for each immunocytochemistry protocol. Additionally, fluorescence thresholds were established from each protocol’s no stain and secondary antibody only controls to exclude background fluorescence when creating binary layers during analysis with the Nikon NIS-Elements software. Expression for each marker was measured by the software as the average area (μm^2^) expressing fluorescence signal.

All analyses were run on unaltered images and representative images chosen for this manuscript have had lookup tables applied within the Nikon NIS-Elements software to brighten signal and were post-processed in Adobe Photoshop (version 22.0; San Jose, CA) to further increase color brightness for printing purposes. All alterations were uniformly applied to the entire image.

### 2.8 Statistics

Statistical analysis was performed using R Statistical Software (version 4.0.3, R Foundation for Statistical Computing, Vienna, Austria) and RStudio (version 1.3.1093, Boston, MA). Statistical models were built using the mixed model function “lme” from package “nlme” (Pinheiro et al., 2021) or ANOVA using the linear model function “lm” (R Core Team, 2020) and *post-hoc* tests were performed using the package “emmeans” (Lenth, 2021) with a Tukey adjustment for multiple comparisons.

## 3 Results

### 3.1 MDM phenotype following vitamin D_3_ treatment

**Figure 1:**
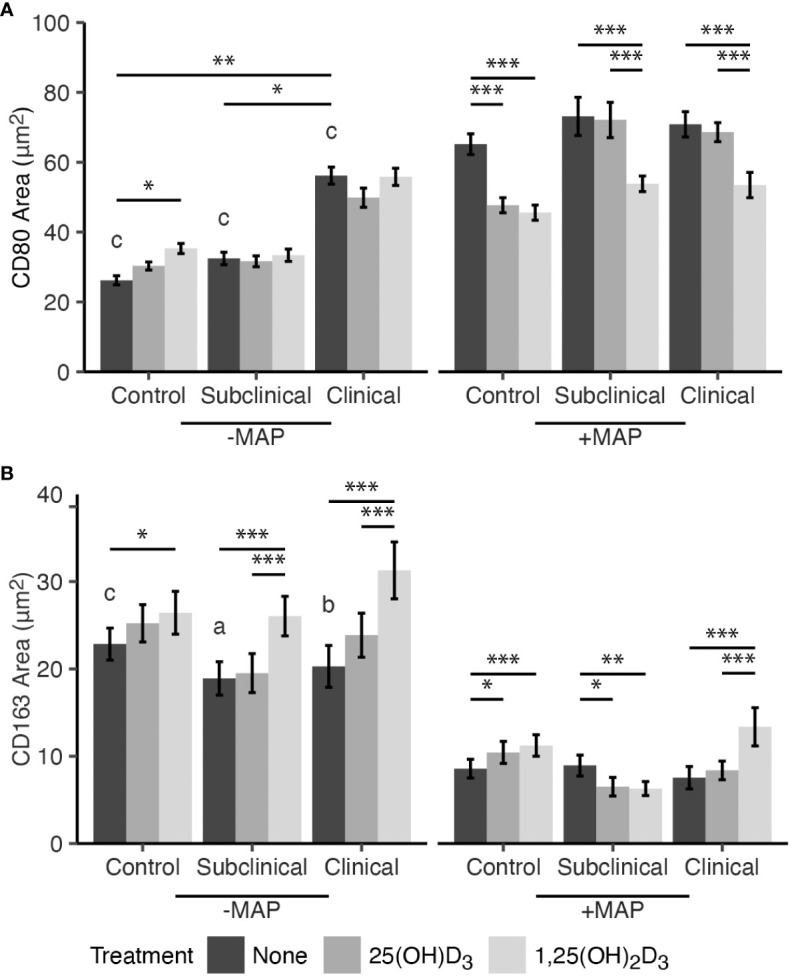
Mean fluorescence area of M1/M2 macrophage phenotype markers **(A)** CD80 and **(B)** CD163. Peripheral blood mononuclear cells (PBMCs) were isolated from the whole blood of dairy cattle naturally infected with *Mycobacterium avium* subsp. *paratuberculosis* (MAP) (JD+ clinical n=7, JD+ subclinical n=7) or JD- controls (n=9). Cells were cultured 5-6 days to generate monocyte-derived macrophages (MDMs), pre-treated with vitamin D_3_ as detailed in methods, then incubated 24 hrs +/- 10:1 MOI live MAP +/- 25(OH)D_3_ or +/- 1,25(OH)_2_D_3_. Expression of CD80 and CD163 were detected using a marker specific primary antibody directly conjugated to AF488 and AF594, respectively. Data are presented as the mean fluorescence area ± SE and significance levels are as follows: * < 0.05, ** < 0.01, *** < 0.001. Comparisons between MAP treatment within JD infection status groups are a < 0.05, b < 0.01, c < 0.001.

The effects of exogenous vitamin D_3_ treatment on macrophage polarization markers CD80 and CD163 following infection with MAP for 24 hrs *in vitro* were assessed using immunocytochemistry and confocal microscopy. 1,25(OH)_2_D_3_ significantly reduced expression of M1 marker CD80 in MAP infected MDMs from JD-, JD+ subclinical, and JD+ clinically infected cows (*P* < 0.001; **Figure 1A**). Additionally, 25(OH)D_3_ reduced CD80 expression only in JD- control cows exposed to MAP *in vitro* (*P* < 0.001). Significant upregulation of CD80 for all JD infection status groups was observed following infection of MDM cultures with MAP (*P* < 0.001). In cell cultures not infected with MAP *in vitro*, the JD+ clinical group had greater expression of CD80 compared to JD+ subclinical cows (*P* < 0.05) and JD- controls (*P* < 0.01). Furthermore, negligible effects of exogenous vitamin D_3_ on noninfected MDMs from each JD status were observed, with only a slight increase in CD80 expression observed for JD- cows upon incubation of MDMs with 1,25(OH)_2_D_3_ (*P* < 0.05).

Expression of M2 phenotype marker CD163 was investigated (**Figure 1B**). Treatment of MDMs with 1,25(OH)_2_D_3_ and infection with MAP *in vitro* resulted in increased CD163 expression in JD- control (*P* < 0.001) and JD+ clinical cows (*P* < 0.001). Treatment with 25(OH)D_3_ also increased expression, but only in the JD- group (*P* < 0.05), and had no significant impacts on MDMs from JD+ clinical animals. In contrast, MDMs from JD+ subclinical animals infected with MAP showed decreased CD163 expression following both 25(OH)D_3_ (*P* < 0.05) and 1,25(OH)_2_D_3_ treatment (*P* < 0.01). Noninfected MDMs from this JD+ group had increased CD163 receptor expression following treatment with 1,25(OH)_2_D_3_ (*P* < 0.001), and similar observations were made in JD- controls (*P* < 0.05) and JD+ clinical cows (*P* < 0.001). Treatment with 25(OH)D_3_ had no significant impacts on any of the JD infection status groups not infected with MAP *in vitro*. Overall, CD163 expression was significantly downregulated following 24 hr MAP infection for MDMs not treated with vitamin D_3_ from JD- controls (*P* <.001), JD+ subclinical (*P* <.05), and JD+ clinically infected animals (*P* <.01).

### 3.2 Vitamin D receptor expression

**Figure 2:**
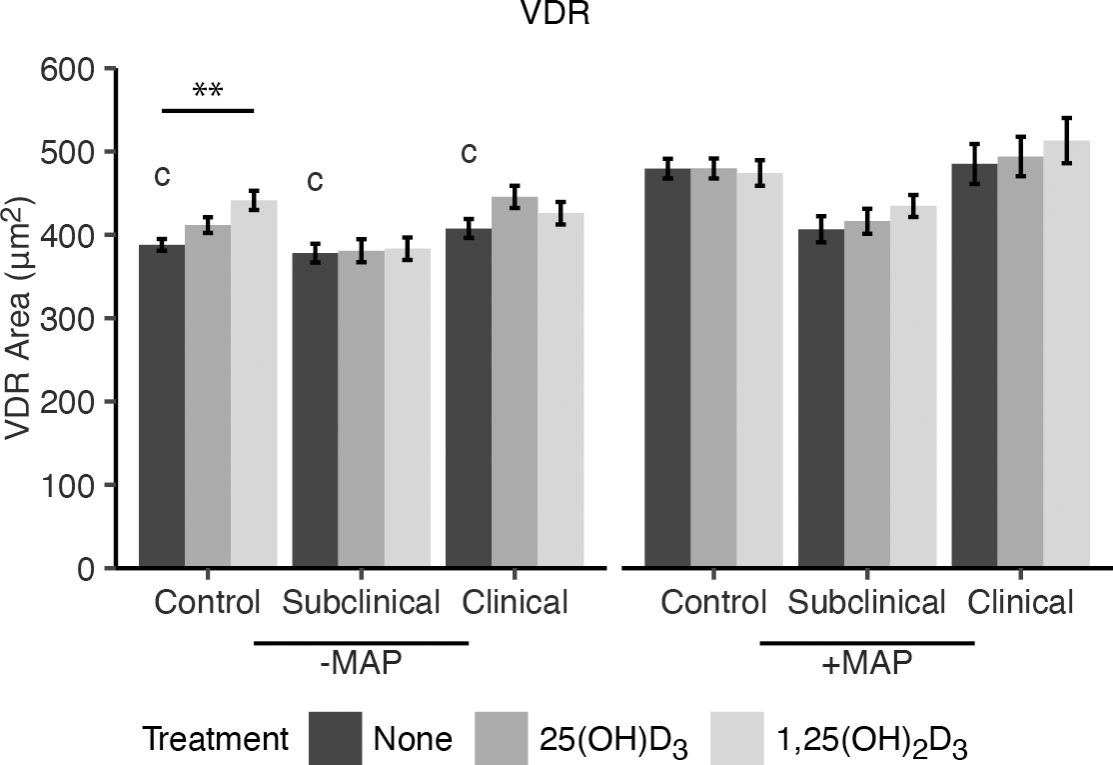
Mean fluorescence area of VDR expression. Peripheral blood mononuclear cells (PBMCs) were isolated from the whole blood of dairy cattle naturally infected with *Mycobacterium avium* subsp. *paratuberculosis* (MAP) (JD+ clinical n=7, JD+ subclinical n=7) or JD- controls (n=9). Cells were cultured 5-6 days to generate monocyte-derived macrophages (MDMs), pre-treated with vitamin D_3_ as detailed in methods, then incubated 24 hrs +/- 10:1 MOI live MAP +/- 25(OH)D_3_ or +/- 1,25(OH)_2_D_3_. VDR expression was detected using a marker specific primary antibody coupled with an AF647 secondary antibody. Data are presented as the mean fluorescence area ± SE and significance is indicated as ** < 0.01. Comparisons between MAP treatment within JD infection status groups are c < 0.001.

**Figure 3:**
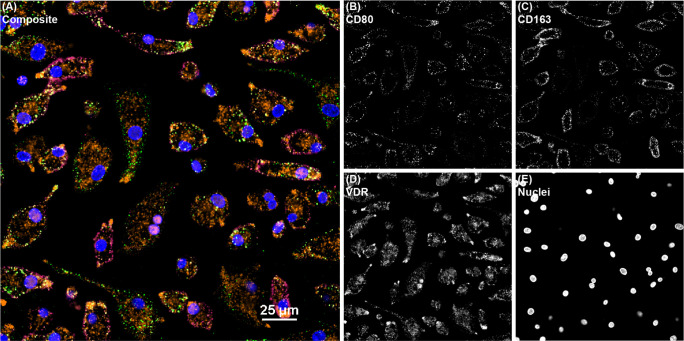
Confocal microscopy image of bovine monocyte-derived macrophages (MDMs) from a JD+ clinical cow treated with 1,25(OH)_2_D_3_ and labeled with markers CD80, CD163, and VDR. All channels are overlayed and shown in the **(A)** composite panel. **(B)** CD80 (green) was directly conjugated to AF488 and excited on the 488 nm laser, while **(C)** CD163 (magenta) was directly conjugated to AF594 and excited on the 561 nm laser. **(D)** VDR (orange), which overlaps as pink in the nuclei, was labeled with an AF647 secondary antibody and excited on the 640 nm laser. **(E)** DAPI counterstain was used to detect nuclei (blue) and was excited on the 405 nm laser.

To elucidate if the effects of vitamin D_3_ on macrophage phenotype markers were a result of increased availability of vitamin D receptor (VDR), expression was measured following addition of either 25(OH)D_3_ or 1,25(OH)_2_D_3_ to MDM cultures (**Figure 2**). Overall, no significant effects due to vitamin D_3_ treatment were observed in MDMs from cows in any JD infection status group following *in vitro* infection with MAP. However, noninfected MDMs from JD- animals that were treated with 1,25(OH)_2_D_3_ did show upregulation of VDR expression (*P* < 0.01). A significant increase in VDR was seen for MDMs not treated with vitamin D_3_ from each infection status group following *in vitro* MAP infection (*P* < 0.001). Confocal microscopy shows expression of CD80 and CD163 localized to the cellular membrane (**Figure 3**). Furthermore, VDR expression can be found localized in higher concentrations within the MDM nuclei and also diffusely expressed in the cytoplasm.

### 3.3 Impact of vitamin D_3_ on intracellular MAP viability

**Figure 4:**
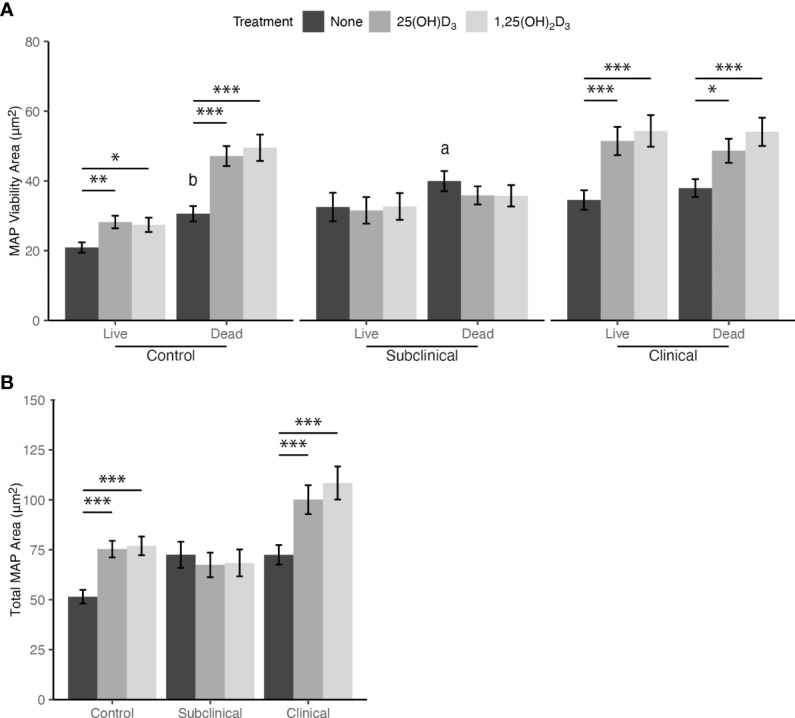
**(A)** Viability and **(B)** total amount of *Mycobacterium avium* subsp. *paratuberculosis* (MAP) phagocytized by monocyte-derived macrophages (MDMs) cultured from naturally infected dairy cattle (JD+ clinical n=7, JD+ subclinical n=7, and JD- controls n=9). Peripheral blood mononuclear cells (PBMCs) were cultured 5-6 days to generate MDMs, pre-treated with vitamin D_3_ as detailed in methods, then incubated 24 hrs with live MAP at 10:1 MOI *+/-* 25(OH)D_3_ or *+/-* 1,25(OH)_2_D_3_. Live and dead MAP were determined using SYTO 9 and propidium iodide, respectively. Data are presented as the mean fluorescence area ± SE. Significant comparisons within vitamin D treatments are * < 0.05, ** < 0.01, *** < 0.001. Comparisons between MAP viability are a < 0.05, b < 0.01.

Intracellular MAP viability was measured using the *Bac*Light assay kit containing P.I. and SYTO 9. A significant increase in live and dead MAP was observed within MDMs from JD+ clinical cows following treatment with 1,25(OH)_2_D_3_ (*P* < 0.001; **Figure 4A**). Similarly, live and dead MAP also increased for this group as a result of 25(OH)D_3_ treatment (*P* < 0.001 and *P* < 0.05, respectively). There were no observed differences between live and dead MAP in MDMs from JD+ clinical cows not treated with vitamin D_3_.

Similar to JD+ clinical cows, increases in the amount of live and dead MAP within MDMs from JD- control cows were observed following treatment with 25(OH)D_3_ (*P* < 0.01 and *P* < 0.001, respectively) and 1,25(OH)_2_D_3_ (*P* < 0.05 and *P* < 0.001, respectively). JD- cows also had a significant increase in dead MAP that was not associated with vitamin D_3_ treatment when compared to live MAP (*P* < 0.01). JD+ subclinical animals showed no significant differences in MAP viability resulting from vitamin D_3_ treatment; however, MDMs not treated with vitamin D_3_ did show higher levels of dead MAP compared to live (*P* < 0.05).

Collectively, the increase in both live and dead MAP following 25(OH)D_3_ or 1,25(OH)_2_D_3_ treatment of MDMs from JD+ clinical and JD- control cows resulted in significantly greater total MAP phagocytized compared to untreated MDMs for both groups (*P* < 0.001; **Figure 4B**). Vitamin D_3_ treatment did not result in any significant changes in MAP phagocytosis for MDMs from JD+ subclinical animals. Additionally, there were no significant differences among MDMs not treated with vitamin D_3_ from any JD status group.

### 3.4 Impact of vitamin D_3_ on endosomal maturation

**Figure 5:**
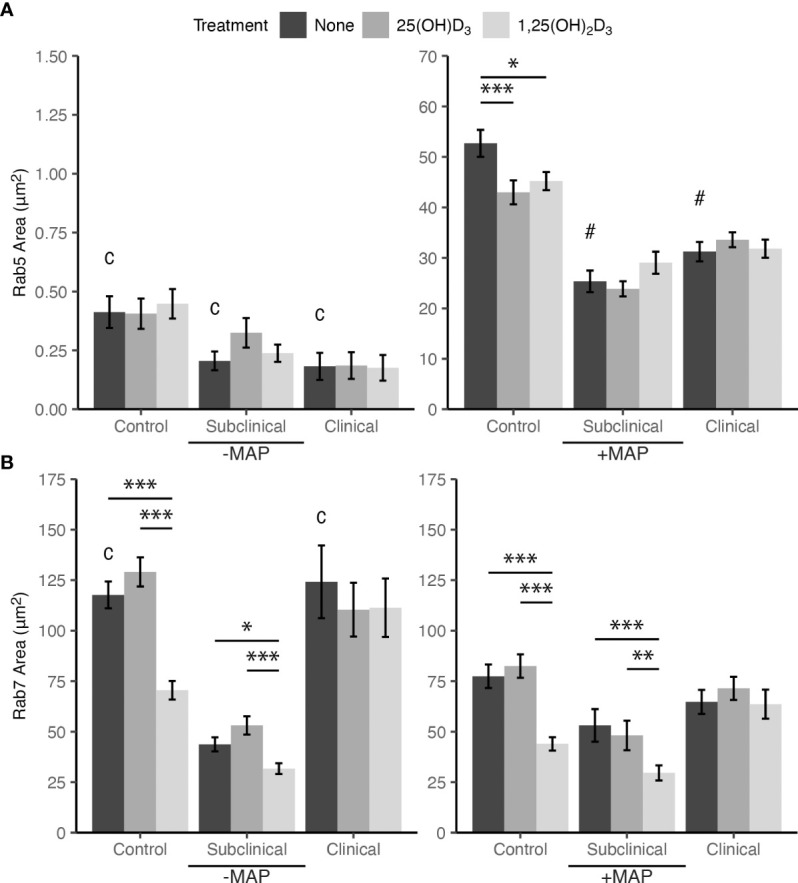
Mean fluorescence area of endosomal maturation markers **(A)** Rab5 and **(B)** Rab7. Peripheral blood mononuclear cells (PBMCs) were isolated from the whole blood of dairy cattle naturally infected with *Mycobacterium avium* subsp. *paratuberculosis* (MAP) (JD+ clinical n=7, JD+ subclinical n=7) or JD- controls (n=9). Cells were cultured 5-6 days to generate monocyte-derived macrophages (MDMs), pre-treated with vitamin D_3_ as detailed in methods, then incubated 24 hrs +/- 10:1 MOI live MAP +/- 25(OH)D_3_ or +/- 1,25(OH)_2_D_3_. Rab5 and Rab7 primary antibodies were both rabbit polyclonal so labeling for each marker was performed in a separate panel. Rab5 primary antibody was coupled with an AF594 secondary and an AF647 secondary antibody was used to detect Rab7. Intracellular labeling was measured by colocalization with macrophage marker CD68 detected with an AF488 secondary antibody. Data are presented as the mean fluorescence area ± SE and significance levels are as follows: * < 0.05, ** < 0.01, *** < 0.001. Comparisons between MAP treatment within JD infection status groups are c < 0.001 and intra-JD status comparisons are # < 0.001.

Markers of endosomal trafficking and maturation were investigated for MAP infected MDMs by fluorescent antibody labeling and confocal microscopy. Rab5 was selected as an early endosomal marker and Rab7 as a marker of late endosomal maturation. MDMs from JD+ subclinical and JD+ clinical cows infected with MAP and not treated with vitamin D_3_ showed significantly lower Rab5 expression when compared to JD- controls (*P* < 0.001; **Figure 5A**). There were no significant differences between JD infection status groups for MDMs not infected with MAP. Additionally, MDMs not infected with MAP also showed no significant effects resulting from vitamin D_3_ treatment within any JD status group, but upon MAP infection Rab5 was significantly upregulated for untreated MDMs from all groups (*P* < 0.001). *In vitro* MAP infection and treatment of MDMs with 25(OH)D_3_ or 1,25(OH)_2_D_3_ resulted in a reduction of Rab5 expression for JD- control cows (*P* < 0.001, *P* < 0.05, respectively). Vitamin D_3_ treatment had no significant effects on MDMs from JD+ cattle.

Collectively, *in vitro* infection with MAP for 24 hrs resulted in significant downregulation of Rab7 expression in MDMs not treated with vitamin D_3_ from the JD- and JD+ clinical groups. JD+ subclinical animals had no significant differences in expression for this comparison. Effects of 1,25(OH)_2_D_3_ treatment on Rab7 expression in MAP infected MDMs from JD- control and JD+ subclinical groups showed a significant reduction compared to each group’s respective untreated MDMs (*P* < 0.001; **Figure 5B**). Decreased expression following 1,25(OH)_2_D_3_ treatment compared to 25(OH)D_3_ treatment was also observed for each of these groups (*P* < 0.001 and *P* < 0.01, respectively). A similar pattern of effects following 1,25(OH)_2_D_3_ treatment was observed for MDMs not infected with MAP. Rab7 expression was downregulated for this treatment in JD- cows compared to 25(OH)D_3_ treatment (*P* < .001) and untreated MDMs (*P* < .001). In addition, JD+ subclinical cows also had decreased Rab7 expression compared to 25(OH)D_3_ treatment (*P* < .001) and untreated MDMs (*P* < .05).

**Figure 6:**
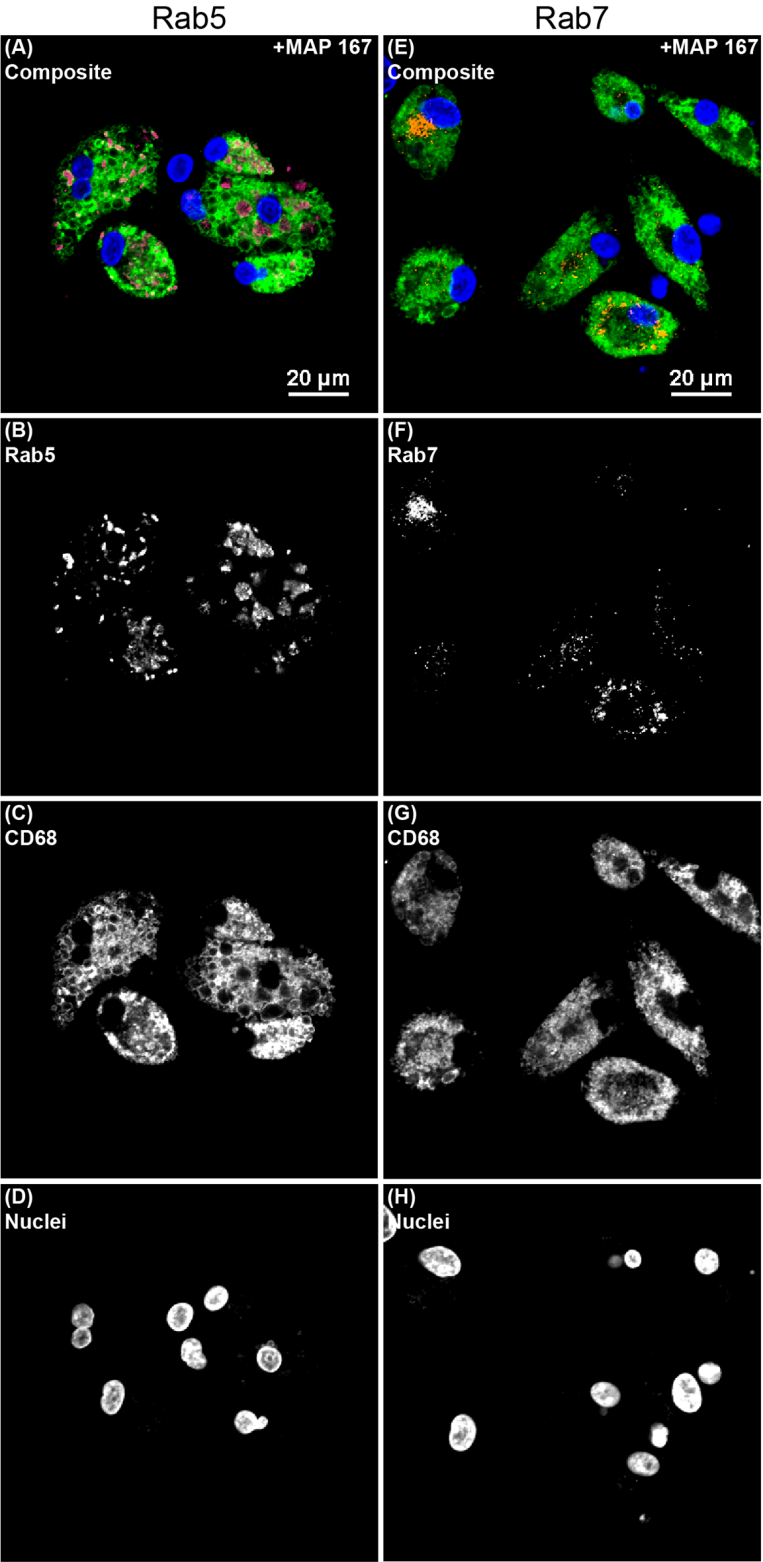
Confocal microscopy image showing endosomal markers within monocyte derived macrophages (MDMs) cultured from a JD- control cow that were infected with *Mycobacterium avium* subsp. *paratuberculosis* (MAP) and treated with 25(OH)D_3_ *in vitro*. Separate protocols were performed for **(A–D)** early endosomal marker Rab5 and **(E–H)** late endosomal marker Rab7. All channels are overlayed and shown in the **(A, E)** composite panels. **(B)** Rab5 (magenta) was labeled with an AF594 secondary antibody and excited on the 561 nm laser, while **(F)** Rab7 (orange) was labeled with an AF647 secondary antibody and excited on the 640 nm laser. **(C, G)** CD68 (green) was labeled with an AF488 secondary antibody and excited on the 488 nm laser. **(D, H)** DAPI counterstain was used to detect nuclei (blue) and was excited on the 405 nm laser.

The degree of change in MDM endosomal marker expression following addition of MAP was vastly different between Rab5 and Rab7. Inoculation of MAP into MDM cultures induced an increase in Rab5 expression by 140× when averaged among all JD status groups (**Figure 5A**). The greatest difference was seen in the JD+ clinically infected group with an increase of 171×. Conversely, the addition of MAP to MDM cultures resulted in an average decrease of Rab7 expression by 1.7× among JD+ clinical and JD- control cows, which were the only two groups with significant changes (**Figure 5B**). **Figure 6** shows endosomal labeling by Rab5 and Rab7 within MAP infected MDMs from a JD- control cow. Labeling of the endosomal markers were performed in independent protocols.

### 3.5 Colocalization of mycobacteria with endosomal markers and LysoTracker

**Figure 7:**
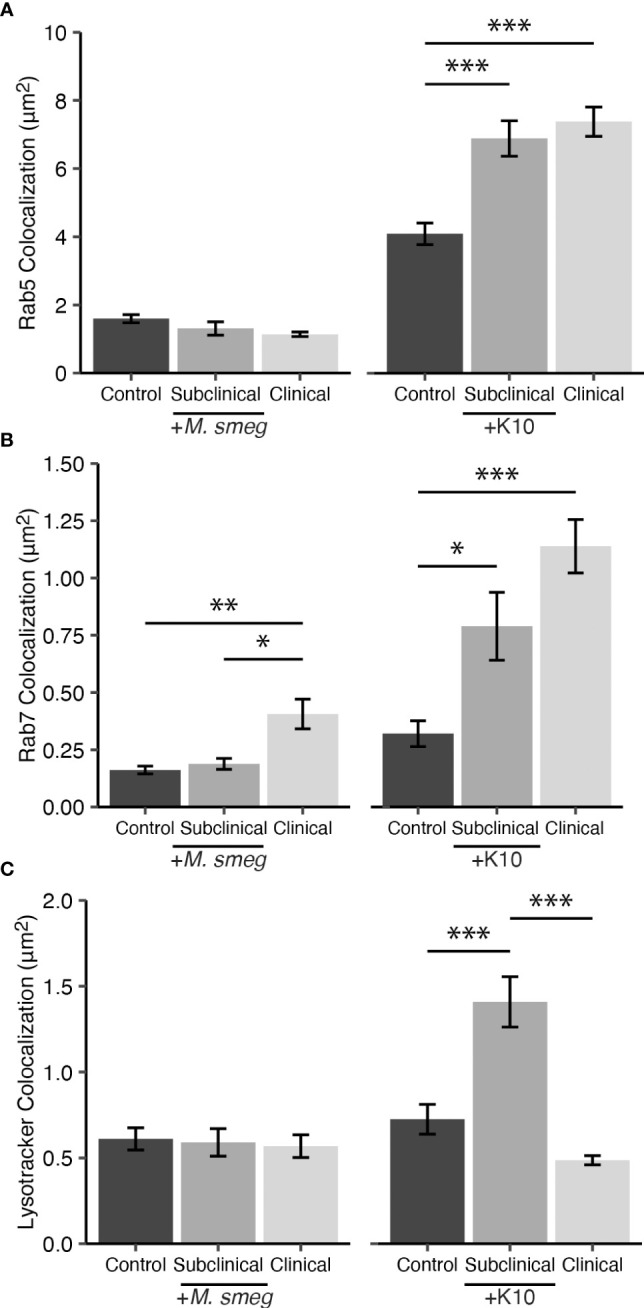
Colocalization of endosomal trafficking markers **(A)** Rab5 and **(B)** Rab7 with SYTO 9 labeled *Mycobacterium smegmatis* (*M. smeg*) and *Mycobacterium avium* subsp. *paratuberculosis* (MAP) K10-GFP within CD68+ monocyte derived macrophages (MDMs). Detection of fluorescent mycobacteria within acidic compartments was measured using **(C)** LysoTracker Red DND-99. A subset of cows naturally infected with MAP (JD+ clinical n=2, JD+ subclinical n=2) or JD- controls (n=2) were inoculated at 10:1 MOI with *M. smeg* or MAP K10-GFP. Data are presented as the mean fluorescence area ± SE and significance levels are as follows: * < 0.05, ** < 0.01, *** < 0.001.

To verify if phagocytized mycobacteria are trafficked to Rab5 and Rab7 containing compartments that could lead to acidification, we utilized MAP K10-GFP as a laboratory-adapted pathogenic strain and environmental non-pathogenic *Mycobacterium smegmatis* (*M. smegmatis*) labeled with STYO 9 to infect MDMs for 24 hrs. Colocalization of bacteria with Rab5, Rab7, and LysoTracker DND-99 were measured on a subset of cows from each JD status group (n=2). Rab5 showed an increase in expression following infection with MAP K10-GFP for both JD+ subclinical and JD+ clinical cows (*P* < 0.001; **Figure 7A**). Colocalization for *M. smegmatis* yielded no significant changes to Rab5. Rab7 had a similar expression pattern to Rab5 following MAP K10-GFP expression, with increased Rab7 expression in JD+ subclinical (*P* < 0.05) and JD+ clinical groups (*P* < 0.001; **Figure 7B**). MDM cultures inoculated with *M. smegmatis* showed Rab7 expression increased only in JD+ clinical cows compared to JD+ subclinicals (*P* < .05) and JD- controls (*P* < .01). JD+ subclinical cows had the greatest amount of LysoTracker and MAP K10-GFP colocalization compared to the JD+ clinical (*P* < 0.001) and JD- control cows (*P* < 0.001; **Figure 7C**). MDM cultures infected with *M. smegmatis* had no differences in bacterial colocalization with LysoTracker. Colocalization for noninfected MDM cultures were undetected since no bacteria are present (data not shown).

## 4 Discussion

Some mycobacterial species are stealthy intracellular pathogens that possess a hallmark characteristic of evading host defensive immune responses. This is achieved through concealment within endosomal compartments that provide niche microenvironments for survival and replication. Mycobacteria modulate host cell apoptosis and arrest maturation of phagosomes transitioning through the endosomal maturation pathway (Clemens et al., 2000a; Hostetter et al., 2003; Kabara and Coussens, 2012). These mechanisms of survival allow for persistence of a chronic subclinical disease state, making the pathogen difficult to eradicate (Windsor and Whittington, 2010).

Work in human pulmonary tuberculosis, caused by *M. tb*, has shown deficient levels of circulating 25(OH)D_3_ is associated with increased susceptibility and severity of disease (Wilkinson et al., 2000; Gibney et al., 2008). Meta-analysis utilizing over 20 studies has reported that humans with tuberculosis infection have significantly reduced serum 25(OH)D_3_ concentrations compared to their healthy counterparts, and 25(OH)D_3_ deficiency is significantly associated with increased risk of infection (Huang et al., 2017). This meta-analysis also included 5 studies that reported individuals with latent tuberculosis infection are at greater risk of developing active infection if they have deficient serum 25(OH)D_3_. Conversely, a more recent meta-analysis including 5 studies suggests relatively high serum vitamin D_3_ levels do not impact the risk of and are not protective against latent tuberculosis infection in humans (Cao et al., 2022). However, the authors state that due to their large confidence intervals further studies need to be included to better assess if there is a relationship between tuberculosis infection and higher vitamin D_3_ levels in the host.

Exposure to 25(OH)D_3_ or its bioactive metabolite 1,25(OH)_2_D_3_ can modulate antimicrobial effects against *M. tb* in humans through induction of autophagy and upregulation of cathelicidin, whose signaling is mediated by retinoid X receptor (RXR) and VDR dimerization followed by binding to vitamin D response elements on genes (Crowle et al., 1987; Liu et al., 2006; Liu et al., 2007; Haussler et al., 2013). The overall lack of significantly different expression of VDR in the present study suggests vitamin D induced effects may be achieved through alternative signaling mechanisms. Further supporting this hypothesis, RXR gene expression has been shown to be reduced in MAP infected macrophages from JD- and JD+ cattle (Ariel et al., 2020). These qPCR results also implicated JD+ status as a significant indicator for reduced RXR expression. Additionally, reduced cholesterol efflux transport pathway activity has been reported, which may result in accumulation of intracellular cholesterol, and is regulated by liver X receptor (LXR)/RXR dimerization (Johansen et al., 2019; Park et al., 2020). A putative modified VDR at the host cell membrane, protein disulfide isomerase family A member 3 (PDIA3), has been shown to bind 1,25(OH)_2_D_3_ and is taken up into the cell through caveolae-mediated endocytosis (Chen et al., 2013; Hu et al., 2019; Zmijewski and Carlberg, 2020). Furthermore, PDIA3 has been shown to associate with nuclear factor κB (NF-κB), a regulator of various pro-inflammatory cytokine responses in the macrophage (Wu et al., 2010). Support for this observation comes from prior work in our lab, where we have shown 1,25(OH)_2_D_3_ to upregulate pro-inflammatory cytokine expression, including IL-1β and IL-12A, in MAP infected MDM-PBMC co-cultures from JD- and JD+ cows at 24 hrs (Wherry et al., 2022a). However, pro-inflammatory IFN-γ and IL-6 were reduced by vitamin D_3_ only in clinical cows, and IL-10 was consistently reduced by vitamin D_3_. More studies are required to fully elucidate alternative vitamin D_3_ signaling pathways in cattle.

Progression of *M. tb* through the macrophage endosomal maturation pathway is regulated through IFN-γ signaling and requires sufficient levels of serum 25(OH)D_3_ in humans (Fabri et al., 2011). While previous work has shown cattle with clinical stage paratuberculosis to have reduced levels of circulating 25(OH)D_3_, physiologic concentrations of 25(OH)D_3_ associated with deficient, insufficient, and therapeutic immune function have yet to be established in cattle (Stabel et al., 2019; Wherry et al., 2022b). 25(OH)D_3_ is the current standard for evaluating vitamin D status of the host, as it is rather stable with a half-life of approximately 15 days (Hollis et al., 2007; Jones, 2008). Conversely, biologically active 1,25(OH)_2_D_3_ has a short (4-6 hr) half-life and is normally found in the circulation in concentrations a 1,000-fold less compared to 25(OH)D_3_ (Jones, 2008). While a concrete range for circulating 1,25(OH)_2_D_3_ has also not been formally established for cattle, it is commonly acknowledged to be present in the picomolar range. Addition of 1,25(OH)_2_D_3_ to cell cultures at 4 ng/ml in the present study is well above probable physiologic serum levels for this vitamin D_3_ analog, but it is reasonable to expect that aggregates of macrophages, such as those in granulomas, could locally produce similar levels (Crowle et al., 1987). Providing further support for this possibility, increased expression of vitamin D_3_ activating hydroxylase CYP27B1 has been shown in monocytes and macrophages following cellular activation (Gyetko et al., 1993; Stoffels et al., 2006; Nelson et al., 2010b).

Vitamin D_3_ contributes to immune responses that promote attenuation and resolution of infectious disease in cattle. Intra-mammary treatment of dairy cattle with 25(OH)D_3_ has shown protective effects in *Streptococcus uberis* (*S. uberis*) induced mastitis (Nelson et al., 2010a; Lippolis et al., 2011). Previous work in our lab has shown treatment with exogenous 25(OH)D_3_ or 1,25(OH)_2_D_3_ facilitates increased phagocytosis of MAP by MDMs co-cultured with PBMCs from JD+ clinically infected cows at 24 hrs following *in vitro* infection (Wherry et al., 2022a). Notably, circulating concentrations of 25(OH)D_3_ in the serum are markedly reduced in clinical cows, possibly as a result of increased intestinal pathology inhibiting nutrient absorption (Stabel et al., 2019). In the present study, treatment of MDMs with 25(OH)D_3_ or 1,25(OH)_2_D_3_ resulted in increased total intracellular MAP, regardless of viability, for JD+ clinical and JD- controls as measured by the *Bac*Light assay. Enhanced phagocytosis induced by vitamin D_3_ has also been observed in a murine macrophage-like cell line model, showing that uptake of yeast cells increases in a dose-dependent manner between 0.2 and 5.0 ng/ml 1,25(OH)_2_D_3_ (Goldman, 1984). This dosage range is similar to the 4 ng/ml used in the present study, but the increased phagocytosis observed by Goldman was not evident until cell cultures reached 45 hrs whereas the present study used a 24 hr timepoint. The addition of 25(OH)D_3_ for the same 45 hr timepoint experiment performed by Goldman also enhanced phagocytosis of yeast cells, but effects were observed only at a high concentration of 500 ng/ml, five times greater than the concentration used in our study. Additional studies in human macrophages and monocytes have reported increased phagocytic activity of *Staphylococcus aureus, Candida albicans* (Small et al., 2021), complement opsonized *Escherichia coli* (Xu et al., 1993), and fluorescent microspheres following vitamin D_3_ treatment (Tokuda and Levy, 1996).

Macrophage activation leads to phenotypic changes that follow the conventional nomenclature denoted as (M1) classically activated or (M2) alternatively activated. M1 macrophages are known for their pro-inflammatory and microbicidal activity, while in contrast the M2 phenotype functions more in resolution and repair and contributes less to infection control. Common markers associated with M1 macrophages include CD80, CD86, and MHCII, while M2 macrophages frequently express CD163 and CD206 (Buechler et al., 2000; Mosser and Edwards, 2008; Ambarus et al., 2012; Khan et al., 2019). In the present study, significant differences due to cow JD infection status were not observed for either CD80 or CD163 following addition of MAP when using the MDM model to differentiate macrophage phenotype. In noninfected MDMs, our study showed a significantly higher amount of CD80 expression in JD+ clinical cows, and no significant differences among JD infection status groups for CD163 expression. To rule out our unfractionated PBMC culture model impacting quantity of MDMs present and skewing results, a mixed model was performed on the number of cells (ROIs) counted for each cow within each JD status. Results showed no significant differences between JD status groups (data not shown). Furthermore, a previous study evaluating monocytes from cattle experimentally infected with MAP reported monocytes from JD+ cattle expressed greater levels of CD80, but had no significant differences in CD163 expression compared to JD- control cows (Thirunavukkarasu et al., 2015). However, when this study stratified animals based on MAP antigen specific IFN-γ production in whole blood, a reduction in CD163 expression was observed in JD+ animals with high IFN-γ expression, which is potentially a representation of subclinical infection, when compared to low IFN-γ responders and JD- control cows. These observations partially align with the present study, which saw a significant reduction in CD163 expression on MDMs from JD+ cattle compared to JD- controls. Discrepancies may be partially explained by the differences in cell types used and nature of host MAP infection methods.

Our investigation into M1/M2 phenotype marker expression in noninfected MDMs revealed vitamin D_3_ modulates expression of these receptors. MDMs from JD- animals treated with 1,25(OH)_2_D_3_ showed a mild increase in both CD80 and CD163 expression. An associated increase was not observed in unstimulated MDMs from JD+ animals for CD80 expression; however, 1,25(OH)_2_D_3_ induced an even greater degree of CD163 upregulation in unstimulated MDMs from JD+ cows. 1,25(OH)_2_D_3_ has been shown to modulate CD163 expression in THP-1 cells, with the greatest degree of gene expression being upregulated when the cells were concurrently activated with BCG (Hsu et al., 2013). BCG alone also significantly increased CD163 expression, while 1,25(OH)_2_D_3_ alone did not. Differences in antigen pathogenicity and measurement of transcript vs protein expression may account for the discrepancies observed in the present study. Additionally, human macrophages differentiated by IL-10 have shown CD163 expression is greatly induced, as opposed to negligible CD163 expressed on pro-inflammatory IL-15 differentiated macrophages (Kim et al., 2018). This may indicate IL-10 played a role in regulating CD163 expression in the current study, however, further studies are needed to analyze concurrent cytokine production and macrophage phenotype receptor expression before this can be confirmed. Regarding cellular function, macrophages possessing a more M2-like phenotype have been associated with enhanced phagocytic capacity (Verreck et al., 2004; Leidi et al., 2009; Sica and Mantovani, 2012; Piñeros et al., 2017). In the present study, the reduction in M1 marker CD80 and increase in M2 marker CD163 following vitamin D_3_ treatment had an associated increase in MAP phagocytosis in JD- and JD+ clinical animals, which may indicate that vitamin D_3_ fostered a more M2-like phenotype in the bovine MDMs. Another study has shown pro-inflammatory IL-1β differentiated human macrophages express significantly more CD163 and have greater phagocytic activity compared to TLR2/1 induced macrophages (Schenk et al., 2014). These studies highlight the complexity of specialized macrophage differentiation and the nuance required to understand the resulting effects on macrophage phenotype and function.

The MAP-induced increase in CD80 and decrease in CD163 expression in the present study for all JD infection status groups may indicate these MDMs overall are more aligned with an M1-like phenotype at this 24 hr timepoint. Differential expression of genes between non-infected and MAP infected macrophages from JD- cattle at 24 hr has shown MAP infection results in upregulated CD80 gene expression and downregulated CD163 (Ariel et al., 2020). These observations align with results in the present study; however, macrophages from JD+ cattle did not differentially express these M1/M2 markers, perhaps because the authors did not parse out responses from subclinical vs clinically infected cattle. There has been some discussion of MDMs developing a hybrid M1/M2 expression profile following pathogen infection or exposure to pro-inflammatory stimuli. Such an expression profile has been recently reported using an *M. tb* infected MDM model and is speculated to contribute to *M. tb*’s intracellular survival (Rao Muvva et al., 2020). Furthermore, monocyte receptor expression profiles are implicated in influencing macrophage phenotype and effector functions following differentiation induced by neutrophil degranulation products (Hussen et al., 2016; Hussen and Schuberth, 2017). Intermediate to high CD14 expression coupled with little to no CD16 was shown to differentiate to a hybrid macrophage phenotype with common M2 associated receptors (increased CD163 and low MHCII) (Hussen et al., 2016). These hybrid MDMs produced higher levels of anti-inflammatory IL-10 but showed competence in controlling infection through increased production of pro-inflammatory IL-12, reactive oxygen species, and enhanced phagocytosis, which contrasts the previous *M. tb* study.

In an effort to further characterize the increased phagocytosis of MAP noted for JD- and JD+ clinical cows, we measured endosomal trafficking and phagolysosome maturation of MDMs. These measures are key factors in survivability of mycobacteria and although well documented in cases of *M. tb* in human models (Upadhyay et al., 2018), much remains to be elucidated in ruminant paratuberculosis.

Results from our study showed *in vitro* infection with MAP induced significant upregulation of Rab5 expression for all JD status groups. Additionally, MDMs from JD+ cattle experience a significant reduction in Rab5 compared to JD- cows upon *in vitro* MAP infection, perhaps indicating that prior MAP exposure dampens Rab5 recruitment or expression. In contrast, a proteomics analysis of ileal tissue in sheep demonstrated increased Rab5 expression in MAP infected animals compared to controls (Pisanu et al., 2018). Additionally, this sheep tissue study reported no significant changes in Rab7 expression between infected and control animals, aligning with observations in our study showing no significant changes between cow infection status groups for Rab7 expression. Pre-incubation of J774 cells with *M. bovis* BCG has been shown to reduce Rab7 recruitment and increase levels of Rab5 when measuring these markers on vesicles containing latex beads (Via et al., 1997). Rab5 acquisition also increased with time from 1 hr to 168 hr after infection with *M. bovis* BCG. Similar observations of increasing Rab5 and decreasing Rab7 expression were significant in our study following addition of MAP to MDMs; however, Rab7 reduction was only significant for JD- and JD+ clinically infected cattle. It is plausible that since IFN-γ promotes the progression of endosomal maturation (Ghigo et al., 2002; Fabri et al., 2011), the stable Rab7 expression in JD+ subclinical animals between vitamin D_3_ and MAP treatments may be related to the fact that cows in this group consistently tested positive during the Bovigam diagnostic analysis, whereas the JD- control group was negative and the majority of the JD+ clinical group tested negative but had some high value positive outliers. Together, these results may provide evidence that in cases of bovine paratuberculosis, Rab5 and/or effectors immediately following infection are the main targets utilized to arrest endosomal maturation and result in reduction of late endosomal marker expression, as is also seen in human *M. tb* infection previously discussed.

To confirm the presence of MAP in the phagosome/lysosome, the present study utilized a smaller subset of cows (n=2) to assess colocalization of mycobacteria with endosomal markers Rab5, Rab7, and LysoTracker-labeled acidic compartments following MDM infection with two alternative mycobacterial species. While investigating specific differences in endosomal trafficking among mycobacterial species was beyond the scope of this study, some interesting observations were presented in these data. Collectively, this experiment did affirm the ability of MDMs to express Rab5 and Rab7 upon infection with mycobacteria, revealing higher levels for JD+ cows, suggesting re-exposure to MAP antigen elicited a trained or memory-like response resulting in heightened activation of the MDM (Kleinnijenhuis et al., 2012; Netea et al., 2016). Although the expression patterns of endosomal trafficking markers in this experiment did not align with earlier results in **Figure 5**, there are notable distinctions between these experiments. Foremost, direct colocalization of Rab5, Rab7, and LysoTracker with mycobacteria within CD68+ MDMs was measured in the **Figure 7** mechanistic experiment, while the results presented in **Figure 5** measured expression of Rab5 and Rab7 throughout the MDM by colocalization with CD68. Additionally, virulence characteristics of the MAP 167 strain and MAP K10-GFP likely contributed to the differences observed. Similar dynamics of mycobacterial virulence have been reported in latent tuberculosis, where the dormant mycobacterium is more immunogenic, loses the ability to express virulence factors that block phagosomal maturation, and is ultimately destroyed more efficiently by the host immune system (Lee et al., 2008; Mariotti et al., 2013). JD+ clinical cows had significantly reduced LysoTracker colocalization with MAP K10-GFP compared to the JD+ subclinical group. Differences in host immunity such as lower levels of IFN-γ and circulating 25(OH)D_3_ may contribute to reduced acidification of mycobacteria-containing compartments (Fabri et al., 2011; Stabel et al., 2019; Wherry et al., 2022b), characteristics that indicate clinical animals may be disproportionately affected and could help explain the impaired destruction of MAP167 and MAP K10-GFP observed in this group. Additionally, humans with low or deficient levels of 25(OH)D_3_ have been reported to have impaired monocyte function (Liu et al., 2006; Adams et al., 2009). While contrasting levels of LysoTracker and Rab7 expression were shown between MAP K10-GFP infected MDMs from JD+ subclinical and JD+ clinical cows, it is important to note that infection with pathogenic mycobacterial species has shown that acquisition of Rab7 is not always an indicator of a phagosome’s progression to acidification and destruction of its contents, as these bacteria can interrupt signaling and binding of effectors downstream from Rab7 (Clemens et al., 2000b; Sun et al., 2007; Keown et al., 2012). In summary, these observations indicate MDM function may be impacted by a combination of factors including host JD status, vitamin D status, and potential mycobacterial strain-specific mechanisms of intracellular survival.

The ability of mycobacterial species to interrupt multiple events in the endosomal maturation pathway, as discussed previously, is a strategic duplication of efforts in response to its immediate environment to ensure its survival. Cows with clinical paratuberculosis face extensive challenges in combating high MAP load while experiencing dampened pro-inflammatory cytokine responses and inhibited nutrient absorption from the diet. Collectively, the present study points to exogenous vitamin D_3_ treatment promoting expression of macrophage markers that coincide with an M2-like phenotype. Vitamin D_3_ afforded JD+ clinical and JD- control cows the ability to upregulate phagocytosis, although JD- cows killed intracellular MAP more efficiently, observing a 58% increase in dead MAP and 33% increase in live MAP. JD+ clinical cows saw this benefit abrogated (35% and 53%, respectively). Previous exposure to high loads of MAP may induce monocyte/macrophage epigenetic changes resulting in dampened protective immunologic responses by the host. Epigenetic changes in monocytes have been reported following vaccination with BCG and these cells obtained “trained immunity” mediated through NOD2 signaling, an intracellular bacterial sensor, affording them a more robust antimicrobial response to non-mycobacterial pathogens (Kleinnijenhuis et al., 2012). One study has shown that NOD2 is not differentially expressed in macrophages from JD- or JD+ cattle (Ariel et al., 2020); however, NOD2 polymorphisms have been associated with a significant increase in susceptibility to MAP infection (Pinedo et al., 2009; Ruiz-Larrañaga et al., 2010).

Interestingly, exogenous vitamin D_3_ maintained expression of Rab5 and Rab7 in JD+ clinical cows. As before, this may indicate previous exposure to high MAP load has downstream effects, maintaining functional expression of these markers in order to “clean up” extracellular MAP, but ultimately resulting in further disease perpetuation by MAP succeeding in obstructing host killing mechanisms. Furthermore, the consistent expression of Rab5 in JD+ cows across treatments may show vitamin D_3_ helps maintain early endosomal marker expression following MAP re-exposure. Another contributing factor could involve vitamin D_3_ signaling through the previously discussed membrane VDR PDIA3. In human gastric epithelial cells, 1,25(OH)_2_D_3_ has been shown to restore calcium-dependent lysosomal acidification, effectively boosting lysosomal degradation capability and allowing for clearance of *Helicobacter pylori* infection (Hu et al., 2019). Vitamin D_3_ treatment effects observed and discussed herein for JD+ clinical cows could be dependent upon exogenous sources of vitamin D_3_, as it is plausible for circulating levels of 25(OH)D_3_ to be used up for other critical processes faster than the animal can absorb through its pathologically inflamed gut. Lastly, Rab7 was consistently downregulated by 1,25(OH)_2_D_3_ in JD- and JD+ subclinical cows regardless of *in vitro* MAP infection, with no negative consequences on intracellular MAP viability. This may suggest other innate mediators of bacterial killing are still active and benefit these animals, such as upregulated nitric oxide and pro-inflammatory IL-1β (Eklund et al., 2013; García-Barragán et al., 2018; Wherry et al., 2022a). Conversely, vitamin D_3_ can inhibit phosphatidylinositol 3-kinase (PI3K) signaling activity by VDR-independent mechanisms, which may have downstream negative regulatory effects on NF-κB induced inflammatory cytokines (Liu et al., 2017; Gkotinakou et al., 2020; Zhao et al., 2021). Furthermore, if vitamin D_3_ can inhibit PI3K signaling, perhaps it can also interfere with PI3P mediated fusion of endosomal compartments thus having negative regulatory effects on trafficking markers, such as the 1,25(OH)_2_D_3_-induced reduction of Rab7 observed herein. The data presented in the current study are novel to the field of vitamin D_3_ signaling in the bovine immune system.

In conclusion, this study provides an early timepoint snapshot of vitamin D_3_ activity for bovine MDM phenotypic changes and its effects on the endosomal maturation pathway *in vitro*. Studies detailing the intracellular trafficking of MAP within bovine macrophages are few, and only recently has work been done to elucidate the effects vitamin D_3_ may play at different stages of paratuberculosis. Future studies would benefit from incorporating a broader panel of M1/M2 and endosomal maturation markers to further elucidate direct relationships between MAP infection, vitamin D interventions, and intracellular trafficking dynamics in the bovine macrophage. Assessing vitamin D_3_-PDIA3 signaling pathways and the resulting effects on immune cell function are also necessary. Understanding the role of vitamin D_3_ in host-MAP dynamics is essential to develop potential therapeutics and intervention strategies for this economically significant pathogen.

## Data availability statement

The original contributions presented in the study are included in the article/supplementary materials. Further inquiries can be directed to the corresponding author.

## Ethics statement

The animal study was reviewed and approved by National Animal Disease Center Animal Care and Use Committee.

## Author contributions

Experimental design was conceived by JS, TW, and RD. Experiments and data analysis were performed by TW. First draft manuscript was prepared by TW and all authors contributed to manuscript revisions. All authors have read and approved the manuscript for submission.

## Funding

This study was funded through USDA-ARS CRIS Project 5030-32000-221.

## Acknowledgments

We thank Adrienne Shircliff (National Animal Disease Center Histology and Microscopy Services Unit) and Amy Turner for their technical expertise and assistance in processing samples, as well as Paul Amundson, Sydney Christen, and the rest of the animal caretaker staff for their support during sample collection. We also thank Matt Inbody for his assistance with bacterial culture and Duane Zimmerman for his assistance in preparing vitamin D_3_ stocks.

## Conflict of interest

The authors declare that the research was conducted in the absence of any commercial or financial relationships that could be construed as a potential conflict of interest.

## Publisher’s note

All claims expressed in this article are solely those of the authors and do not necessarily represent those of their affiliated organizations, or those of the publisher, the editors and the reviewers. Any product that may be evaluated in this article, or claim that may be made by its manufacturer, is not guaranteed or endorsed by the publisher.
